# Supplementary material for: Multiscale cardiac imaging spanning the whole heart and its internal cellular architecture in a small animal model
Source: eLife. 2020 Oct 20;9:e58138. doi: 10.7554/eLife.58138 (PMC7595733; doi:10.7554/eLife.58138)
Supplement: Transparent reporting form [file elife-58138-transrepform.docx]

***eLife’s* transparent reporting form**

We encourage authors to provide detailed information *within their submission* to facilitate the interpretation and replication of experiments. Authors can upload supporting documentation to indicate the use of appropriate reporting guidelines for health-related research (see [EQUATOR Network](http://www.equator-network.org/%20)), life science research (see the [BioSharing Information Resource](https://biosharing.org/)), or the [ARRIVE guidelines](http://www.plosbiology.org/article/info:doi/10.1371/journal.pbio.1000412) for reporting work involving animal research. Where applicable, authors should refer to any relevant reporting standards documents in this form.

If you have any questions, please consult our Journal Policies and/or contact us: [editorial@elifesciences.org](mailto:editorial@elifesciences.org).

**Sample-size estimation**

- You should state whether an appropriate sample size was computed when the study was being designed
- You should state the statistical method of sample size computation and any required assumptions
- If no explicit power analysis was used, you should describe how you decided what sample (replicate) size (number) to use

Please outline where this information can be found within the submission (e.g., sections or figure legends), or explain why this information doesn’t apply to your submission:

Our article falls within the category of “Tools and Resources”, thus we did not compute sample sizes. We presented a novel multiscale imaging approach to image the whole heart (4-5 mm wide) with micrometer resolution, and the internal architecture of its cells with nanometer resolution. The objective of the study was to report on our developed protocols, and show they are reproducible. To prove reproducibility (technical replication), we chose to report on images acquired from two samples (a control and a treated sample with a heart defect), as an imaging example only, without trying to infer biological insights. For our study, thus, sample sizes were not computed as such calculation was not needed.

**Replicates**

- You should report how often each experiment was performed
- You should include a definition of biological versus technical replication
- The data obtained should be provided and sufficient information should be provided to indicate the number of independent biological and/or technical replicates
- If you encountered any outliers, you should describe how these were handled
- Criteria for exclusion/inclusion of data should be clearly stated
- High-throughput sequence data should be uploaded before submission, with a private link for reviewers provided (these are available from both GEO and ArrayExpress)

Please outline where this information can be found within the submission (e.g., sections or figure legends), or explain why this information doesn’t apply to your submission:

Because our article is a “Tools and Resources” article, we reported on technical replicates only. After developing the imaging methodology presented, we applied it to (and reported on) two hearts, one normal and one with a defect, and showed that our methodology worked for both hearts. We highlighted this in the introduction, “As proof of concept, we applied our developed multiscale imaging procedure to two embryonic chick hearts.” The images from these two hearts were compared and analyzed in the Results section, and discussed in the Discussion section.

**Statistical reporting**

- Statistical analysis methods should be described and justified
- Raw data should be presented in figures whenever informative to do so (typically when N per group is less than 10)
- For each experiment, you should identify the statistical tests used, exact values of N, definitions of center, methods of multiple test correction, and dispersion and precision measures (e.g., mean, median, SD, SEM, confidence intervals; and, for the major substantive results, a measure of effect size (e.g., Pearson's r, Cohen's d)
- Report exact p-values wherever possible alongside the summary statistics and 95% confidence intervals. These should be reported for all key questions and not only when the p-value is less than 0.05.

Please outline where this information can be found within the submission (e.g., sections or figure legends), or explain why this information doesn’t apply to your submission:

We reported statistics on image quantifications from our two samples. We described the methods employed for quantification and statistical analysis in the “Material and Methods” section under “Image analysis and segmentation”, and results were reported in the “Results” section under “Image segmentation and quantification”, and summarized in Figure 7.

(For large datasets, or papers with a very large number of statistical tests, you may upload a single table file with tests, Ns, etc., with reference to sections in the manuscript.)

**Group allocation**

- Indicate how samples were allocated into experimental groups (in the case of clinical studies, please specify allocation to treatment method); if randomization was used, please also state if restricted randomization was applied
- Indicate if masking was used during group allocation, data collection and/or data analysis

We only had two groups: control and hemodynamically treated embryo hearts. The details on how the embryos were prepared for each group are in the “Materials and Methods” section under “Generation of cardiac defects” and references therein. Within the hemodynamically treated group, we chose to present one embryo with a congenital heart disease. The embryo heart was chosen using micro-CT imaging, described in the “Materials and Methods” section under “Cardiac processing enabling micro-CT imaging”, and further explained in the “Results” section under “Cardiac structure analysis from 3D micro-CT images”.

Please outline where this information can be found within the submission (e.g., sections or figure legends), or explain why this information doesn’t apply to your submission:

**Additional data files (“source data”)**

- We encourage you to upload relevant additional data files, such as numerical data that are represented as a graph in a figure, or as a summary table
- Where provided, these should be in the most useful format, and they can be uploaded as “Source data” files linked to a main figure or table
- Include model definition files including the full list of parameters used
- Include code used for data analysis (e.g., R, MatLab)
- Avoid stating that data files are “available upon request”

Please indicate the figures or tables for which source data files have been provided:

We provide a source data file (excell file) for the data presented in Figure 7. In addition we will upload raw images and segmented images into Dryad website.
